# Incidence, Impact, and Healthcare‐Seeking Behavior for Extremity Fractures in Resource‐Limited Settings: A Household Survey in Rural Tanzania

**DOI:** 10.1002/wjs.12540

**Published:** 2025-04-11

**Authors:** Joost J. Binnerts, Thom C. C. Hendriks, Jovine Okoth, Annelise Gill‐Wiehl, Kavitha Ranganathan, Anam N. Ehsan, Nkaina W. Harun, Shelly Ogoya, Nefti Bempong‐Ahun, Geoffrey Ibbotson, William J. Harrison, Claude Martin, Michael J. R. Edwards, Erik Hermans, Bwire M. Chirangi

**Affiliations:** ^1^ Radboud University Medical Centre Nijmegen The Netherlands; ^2^ Stichting Shirati Amsterdam the Netherlands; ^3^ Slingeland Hospital Doetinchem The Netherlands; ^4^ Shirati KMT Hospital Shirati Tanzania; ^5^ University of California Berkeley University Avenue and Oxford St Berkeley California USA; ^6^ Brigham and Women's Hospital Boston Massachusetts USA; ^7^ Global Surgery Foundation Geneva Switzerland; ^8^ AO Alliance Davos Switzerland

**Keywords:** disability, fracture, healthcare‐seeking behavior, incidence, resource‐limited settings, survey

## Abstract

**Background:**

Limited research exists on the burden of extremity fractures in Sub‐Saharan Africa. Underreporting is likely, as patients often seek out traditional bonesetters (TBSs). This study aims to determine the annual incidence and impact of extremity fractures, alongside health‐seeking behavior of patients in rural Tanzania.

**Methods:**

We conducted a cross‐sectional household survey in Rorya district, Tanzania, enrolling 497 households with 2667 members, using spatial random sampling. We surveyed household heads regarding access to fracture care and fracture occurrence among household members. We then randomly selected up to three members per household to survey, using the 1448 responses to calculate extremity fracture incidence. Any (self‐)reported fractures were questioned on healthcare‐seeking behavior and assessed through radiological evaluation. Confirmed cases completed a survey on disability and financial impact.

**Results:**

We radiologically confirmed 11 extremity fractures among 1448 randomly selected respondents, yielding an annual incidence of 0.76%. Five additional fractures were identified among nonrandomized individuals totaling 16 confirmed cases. TBS attendance among patients suspecting fracture was significantly higher than hospital attendance (95% vs. 32%, *p* < 0.0005). Primary reasons for choosing TBSs were lower cost (62%) and perceived faster healing (29%). Sixty‐two percent of patients reported reduced work capacity or requiring help with transport and 50% experienced a decrease in income.

**Conclusions:**

The annual incidence of extremity fractures in this study was 0.76%. TBSs were largely preferred over hospitals due to lower cost and perceived faster healing. Over half of patients experienced reduced ability to work and income loss. Improved communication between TBSs and hospitals, along with better access to hospital care, could reduce complications.

## Graphical abstract

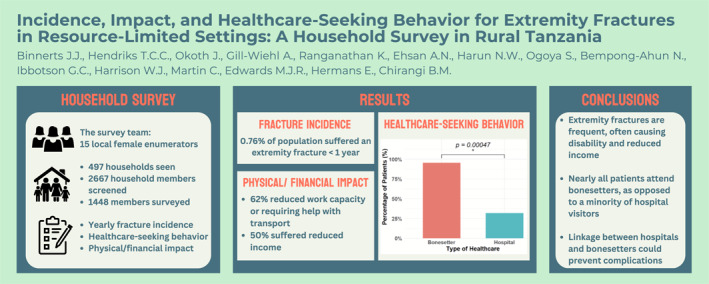

**Abbreviations**
LMIC: Low‐ or middle‐income country
NIMR: National Institute for Medical Research
RTI: Road traffic injury
SFIA: Short Financial Impact Assessment 3
SOSAS: Surgeons OverSeas Assessment of Surgical need
TBS: Traditional bonesetter

## 1 Introduction

Trauma is a major contributor to the global surgical burden and mortality, causing 8% of annual deaths worldwide [1]. This burden is disproportionately high in low‐ and middle‐income countries (LMICs) due to causes such as road traffic injuries (RTIs), interpersonal violence, and conflicts. Among these, RTIs alone account for 1.3 million deaths worldwide each year.

In Africa, 38 per 1000 disability‐adjusted life years (DALYs) are attributable to traumatic injury compared to 27 per 1000 DALYs worldwide. Young adults bear the brunt of these injuries, leading to not only loss of life but also long‐term disability and loss of income. Without intervention, the overall trauma burden is expected to rise in LMICs [1, 2, 3].

In Northern Tanzania, over 90% of the population cannot access orthopedic surgical services [4]. In such settings, this gap is filled by traditional bonesetters (TBSs) [5, 6, 7, 8] due to their cultural acceptability, increased affordability, and geographical convenience [5, 9]. TBSs commonly treat fractures through a combination of fracture massage, splinting, reduction, and herbal medication [10, 11].

Pending the expansion of a web‐based trauma registry used in Tanzania's tertiary orthopedic center Muhimbili Orthopedic Institute [12], national data on the epidemiology and clinical outcome of patients with extremity fractures, especially those treated by TBSs, is still lacking. Some studies have postulated that relatively simple fractures are likely successfully treated by TBSs, yet fractures which require surgical intervention are not adequately addressed [13, 14]. Resulting complications, such as nonunion or malunion, osteomyelitis, or soft tissue gangrene, may require extensive surgery or even amputation [15, 16].

In Tanzania, the level of TBS patronage is unknown, and data regarding the burden of disease of extremity fractures are limitedly available. Previous studies in Tanzania focus on patients with fractures presenting at the hospital or rely on mathematical models [3, 17]. These approaches exclude patients who do not reach the hospital or are treated otherwise, potentially underreporting and consequently underestimating the impact of extremity fractures.

This study aims to provide an estimate of the annual incidence and subsequent impact of extremity fractures in an area with high patronage of bonesetters, using a cross‐sectional household survey in rural Tanzania. We specifically focus on fractures of the extremities because, unlike vertebral fractures, there is a greater potential for optimal treatment in these resource‐limited settings, where advanced surgical options at district hospitals are typically sparse. Additionally, we investigate healthcare‐seeking behavior among suspected fracture patients.

## 2 Materials and Methods

### 2.1 Study Site

We conducted a household survey around Shirati, located in Rorya district, Tanzania, which counts 354,490 inhabitants [18]. Residents largely relied on subsistence agriculture (77%) and had limited access to paved roads, piped water (27.8%), sewage (4%), and grid electricity (6.1%) [19]. In Rorya's district hospital, “traumatic injuries” were the third most common diagnosis in 2021 (unpublished hospital data). The nearest referral orthopedic center is a 5 h car drive to Mwanza. Rorya district is known for its high TBS patronage, likely in part due to its rurality. However, despite government efforts, no reliable data are available on the number of active TBSs in the district.

### 2.2 Study Design

**FIGURE 1:**
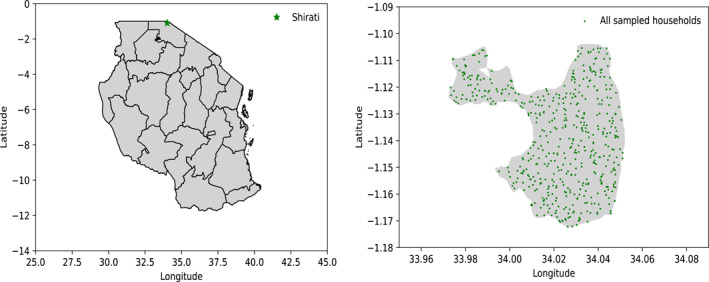
This figure illustrates the random sampling method, which randomly selects points within Shirati. The left panel depicts Shirati within Tanzania. The right panel identifies the randomly selected points within Shirati.

This population‐based study entailed a series of cross‐sectional surveys. We approached a random sample of 511 households, previously identified by Gill‐Wiehl et al. using a spatial random sampling method (see supplemental material) [20]. Google Maps and Python were used to randomly place 511 points within the wider Shirati area, after which locally recruited enumerators, who knew the area well, traveled on foot or using a motorcycle taxi to invite the closest household to participate (Figure 1).

The head of household provided demographic information and access‐to‐care data for the household's members. A household member was defined as a person who ate from the same pot and slept in the same structure the night before. Household members were selected using R [21] to randomly allocate numbers to each household roster, regardless of presence at the time of survey to avoid sex and age selection bias.

We then surveyed the randomly chosen household members to calculate the annual incidence rate of extremity fractures. Subsequently, we approached all household members who either self‐reported an extremity fracture in the last 12 months or were reported by the head of household for radiological confirmation within 2 weeks and administration of a survey on healthcare‐seeking behavior. After obtaining consent, X‐ray images were made using a portable X‐ray imaging system (Delft Ultra, Delft Imaging Systems BV) and interpreted by a certified radiologist. An extremity fracture was defined as any partial or complete break in the continuity of an upper (fingers to clavicle) or lower extremity bone (toes to femur). All confirmed fracture patients answered additional survey questions on disability and financial impact.

### 2.3 Data Collection Tools

We designed three surveys using SurveyCTO [22] on.

1. Access‐to‐care: Administered to household heads, consisting of a household roster and questions relating to health travel, based on the Surgeons OverSeas Assessment of Surgical need (SOSAS). This survey was validated previously in several countries [23, 24].
2. Incidence: Targeted three randomly selected household members, gathering demographic data and extremity fracture incidence <12 months. This mini‐survey was self‐developed and validated during the pilot.
3. Healthcare‐seeking behavior and disability: Administered to suspected fracture patients, collecting data on fracture type, healthcare choices, and remaining disability using the SOSAS instrument and a Short Financial Impact Assessment (SFIA), pertaining to loss of job/income. The SFIA was self‐developed in association with Harvard's Program in Global Surgery and Social Change and validated for reliability in the pilot.

All surveys were translated to and administered in Kiswahili or Kijaluo (see supplemental material).

### 2.4 Survey Team and Pilot Survey

The survey team consisted of 15 local Tanzanian women with prior experience conducting surveys. All survey enumerators received a 5‐day training in questionnaire administration (see supplemental material for the training program). We conducted a pilot study with 123 randomly selected neighboring households and 239 household members to validate the surveys and guide target sample size; however, we were unable to confirm reported fractures radiologically within the pilot as the X‐ray system had not yet passed through customs. The pilot showed good offline performance and satisfactory survey adequacy, with only minor textual changes necessary for clarity.

### 2.5 Sample Size

The pilot yielded 5 extremity fracture patients in the last 12 months out of 239 respondents, suggesting a 2.1% yearly incidence. We determined the target sample size with an accepted margin of error (e) of 1%, a 99% confidence interval (Z) of 2.576, and an estimated yearly incidence of extremity fractures (p) of 0.021 using Equation (1):

(1) $$N=Z^{2}\;*\;p\;*\;\left(1-p\right)\;/\;e^{2}$$

This yielded a sample size of 1365 respondents. We overpowered by 10%, targeting three household members within each of the 511 randomly selected households [25].

### 2.6 Outcome Measures

Our primary outcome was the yearly incidence calculated as the percentage of confirmed extremity fracture patients <12 months among the total number of randomized household members. Healthcare‐seeking behavior outcomes included the proportion of suspected extremity fracture patients who reported attending the hospital and/or TBS as well as main reasons behind this health choice. Finally, we measured disability and financial impact using the SOSAS questionnaire's 5‐point Likert scale and the SFIA questionnaire.

### 2.7 Statistical Analysis

We described household and individual respondent characteristics using central tendency measures, frequencies, and percentages. We tracked the number of confirmed extremity fracture patients in the last 12 months to calculate our primary outcome of interest and annual incidence. We performed an exact McNemar's test to determine if TBS versus hospital attendance was statistically different. Data were analyzed using R software, version 4.3.0.

### 2.8 Ethical Clearance and Patient Protection

Ethical clearance was obtained through the National Institute for Medical Research (NIMR) of Tanzania (NATHREC‐NEW‐2022‐339). Informed consent was obtained and stored digitally. Participants under 18 years required additional caretaker consent. We reiterated the risks of X‐ray imaging to suspected fracture patients and requested additional verbal consent prior to imaging. Any pregnant patients would have been excluded from the X‐ray verification. In agreement with NIMR, respondents were given 5000 Tanzanian Shillings (TSH) (∼2 US Dollar [All TSH to USD conversion as of November 1st, 2023]) for their time.

## 3 Results and Discussion

### 3.1 Participant Characteristics

Between November and December 2023, we surveyed 497 households, comprising a total of 2667 members, with an average household size of 5.4 members (SD 2.03). Fourteen households had moved away since the initial sampling of 511 households. Household members had a median age of 16 years (IQR 9–35) and were balanced across sex.

**FIGURE 2:**
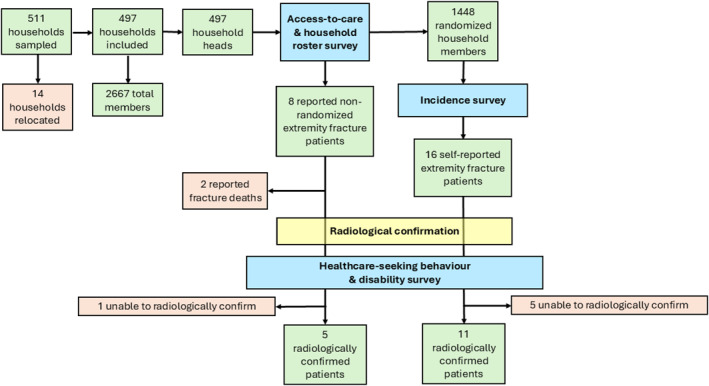
Flowchart describing sampling and survey procedures.

In the incidence survey, a total of 1448 respondents participated, with median age of 17 years (IQR 10–38) and an even split in sexes (Table 1). Sixteen randomized household members reported an extremity fracture in the last 12 months. In addition, six fracture suspects among nonrandomized members were reported by heads of households. Of these 22 suspected fracture patients, the X‐ray verification confirmed 16 cases (Figure 2). One suspected case had suffered a joint luxation instead and five showed no clear signs of fracture.

**TABLE 1:** Randomized household member demographics.

Variable	*N* = 1,448^a^
**Age**	17 (10, 38)
**Sex**	
Male	718 (50%)
Female	730 (50%)
**Level of completed education**	
None (incl. kindergarten)	419 (29%)
Primary education	822 (57%)
Secondary education	187 (13%)
College degree	17 (1.2%)
University degree	3 (0.2%)
**Occupation**	
Unemployed (incl. retirees and students)	842 (58%)
Home maker	156 (11%)
Domestic helper	12 (0.8%)
Farmer/Fisher	302 (21%)
Self‐employed/entrepreneur	123 (8.5%)
Government employee	9 (0.6%)
Nongovernment employee	4 (0.3%)
**Tribe**	
Luo	1346 (93%)
Kurya	20 (1.4%)
Sukuma	16 (1.1%)
Jita	12 (0.8%)
Other	54 (3.7%)
**Extremity fracture reported < 12 months**	
Yes	16 (1.1%)
No	1432 (99%)

^a^ Median (Q1, Q3) and *n* (%).

The 16 confirmed fracture patients were predominantly male (75%) and had a median age of 19 years (IQR 13–33).

### 3.2 Access‐To‐Care

**FIGURE 3:**
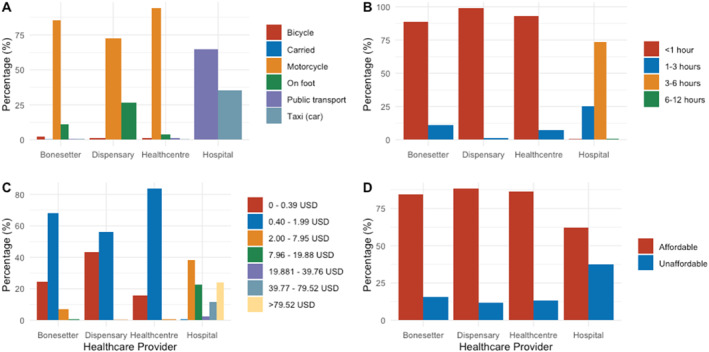
Access to care responses. Panel A shows the distribution of the mode of transport used to reach healthcare providers. Panel B shows the travel time upon seeking healthcare. Panel C displayed the costs associated with travel to healthcare providers. Panel D summarizes self‐reported affordability of travel costs.

Most household heads reported traveling to dispensaries and traditional bonesetters using a motorcycle or on foot; respondents predominantly reached health centers using a motorcycle and underwent hospital visits with public transport and cars (Figure 3). Travel time to dispensaries and bonesetters was generally under an hour, contrasting with the 3–6 h required for hospital visits (Figure 3). Costs to reach dispensaries, health centers, and bonesetters were mostly under 5000 TSH, whereas hospital visits incurred markedly higher travel expenses. Thirty‐seven percent of respondents reported that referrals to hospitals were not affordable (Figure 3).

### 3.3 Annual Incidence

Out of 1448 randomized household members, 16 (1.1%) reported extremity fractures in the past year (Table 1). These participants underwent x‐ray imaging for verification, during which 11 extremity fracture patients were confirmed, yielding an annual extremity fracture incidence rate of 0.76%. Out of the 16 confirmed fractures, forearm fractures were most common (38%), followed by the upper arm (31%). The leading causes of fractures were falls (50%) and motorcycle accidents (44%) (Table 2).

**TABLE 2:** Healthcare‐seeking behavior among patients with suspected fracture.

Variable	*N* = 16^a^
**Age**	19 (13, 33)
**Months since fracture**	6 (5, 7)
**Sex**	
Male	12 (75%)
Female	4 (25%)
**Fracture location**	
Finger/toe	0 (0%)
Hand	0 (0%)
Lower arm	6 (38%)
Upper arm	5 (31%)
Foot	1 (6.3%)
Lower leg	2 (13%)
Upper leg	2 (13%)
**Cause of fracture**	
Other	1 (6.3%)
Car/bus	0 (0%)
Motorcycle accident	7 (44%)
Gunshot	0 (0%)
Violence	0 (0%)
Animal‐related	0 (0%)
Fall	8 (50%)
Explosion	0 (0%)
Fire‐related	0 (0%)
**Disability**	
No disability	6 (38%)
I feel ashamed	0 (0%)
Unable to work like before	9 (56%)
Need help with transport	1 (6.3%)
Need help with daily living	0 (0%)
**Return to work**	
Yes, like before	3 (19%)
Yes, same job, but limited activities	2 (13%)
Yes, different kind of job	1 (6.3%)
No, lost job due to injury	6 (38%)
No, lost job for other reason	0 (0%)
Unemployed/retired at time of injury	4 (25%)
**Work missed**	
None	0 (0%)
Less than 1 day	0 (0%)
1–5 days	0 (0%)
Between 5 days and 1 month	0 (0%)
More than 1 month	12 (75%)
Unemployed/retired at time of injury	4 (25%)
**Income change since injury**	
Income reduced	8 (50%)
Income similar	8 (50%)
Income increased	0 (0%)

^a^ Median (Q1, Q3) and *n* (%).

### 3.4 Healthcare‐Seeking Behavior

**FIGURE 4:**
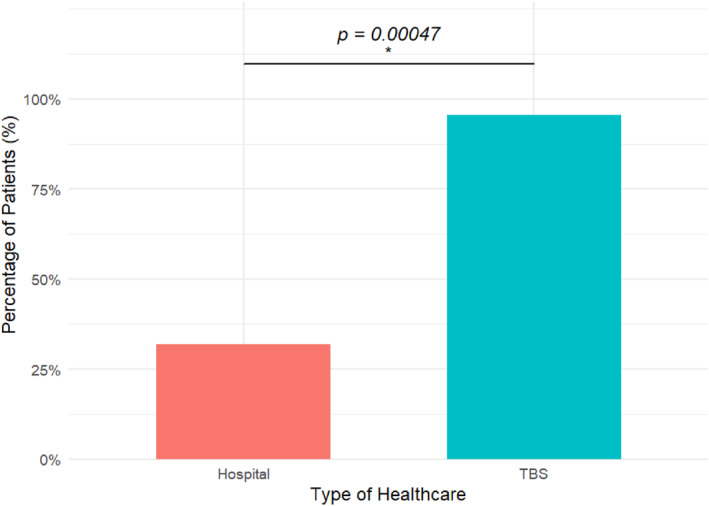
Healthcare‐seeking behavior among confirmed fracture patients. Note: patients could attend both the hospital and the TBS.

Besides the 16 self‐reported fracture suspects among the randomized sample, the heads of household reported 2 extremity fracture‐related deaths as well as 6 nonrandomized household members with a suspected extremity fracture in the last year. Out of these 22 suspected fracture patients, seven (32%) had attended a health facility, whereas a vast majority (95%) had sought treatment from TBSs. The exact McNemar test indicated a statistically significant difference in the probability that a fracture patient attends a TBS compared to a health facility (*p* < 0.0005) (Figure 4). Six patients (27%) had attended both the hospital and a traditional bonesetter. TBS treatments included traditional massage (94%) and traditional splinting (63%). Only 4 out of 7 hospital patients had received plaster of Paris while no fracture surgery was carried out. The primary reasons for choosing TBS were lower cost (62%) and perceived quicker healing (29%) (Table 3).

**TABLE 3:** Fracture‐related characteristics, physical disability, and financial impact among patients with confirmed fracture.

Variable	*N* = 22^a^
**Attended health facility**	7 (32%)
**Attended TBS**	21 (95%)
**Reason for not seeking hospital care**	
No money for treatment	10 (67%)
No money for transport	1 (6.7%)
Bad staff attitude	0 (0%)
Fear/lack of trust	1 (6.7%)
No equipment/skilled staff	0 (0%)
No perceived need	3 (20%)
Given referral, but did not go	0 (0%)
**Reason for seeking traditional care**	
Fear of surgery	1 (4.8%)
Lower cost	13 (62%)
No trust in hospital treatment	0 (0%)
Previous positive TBS experience	1 (4.8%)
Spiritual help	0 (0%)
TBS heals quicker	6 (29%)
Better patient care	0 (0%)

^a^ *n* (%).

### 3.5 Disability and Financial Impact

Thirty‐eight percent of patients reported no disability, whereas 62% indicated being unable to work as before or requiring help with transport. The median time at which respondents were surveyed since injury was 6 months (IQR 5–7).

Regarding return to work, 19% resumed their previous job without limitations, 13% with limited activities, and 6% switched to a different job. Notably, 38% lost their job due to the injury. The duration of work missed due to the injury varied, with 75% missing more than 1 month. Income changes post‐injury showed that 50% experienced a reduction in income, whereas the other half reported no change (Table 2).

## 4 Discussion

Our study employed a community‐based methodology, including random selection at the level of and within the household and utilization of the portable X‐ray imaging for remote fracture confirmation. With this robust methodology, it provides first insights into the incidence of extremity fractures in rural East‐Africa, reporting an annual incidence of 0.76% in Rorya district, Tanzania. Nearly all (95%) of these patients attended a TBS, compared to 32% hospital attendance. Physical (62%) and financial (50%) consequences were common among extremity fracture patients.

The 0.76% incidence rate, although lower than previous estimates, highlights a significant burden of fractures in the study population. Wu et al. estimated a global 2.3% incidence rate of fractures of any type in their study model, whereas Sawe et al. in a one‐day survey across 105 Tanzanian hospital similarly found that 2.2% of patients presented with bone fractures of any type, including those of the spine and ribs [3, 17]. Our narrower focus on extremity fractures, use of the X‐ray verification, and sampling of the general population, instead of a population presenting in the emergency department, may have resulted in the lower observed incidence. Contrarily, a large household survey in Cameroon, relying on self‐reporting without X‐ray confirmation, found a lower annual extremity fracture incidence of 0.43% looking at urban and rural settings [26]. Their lower incidence may be attributed to the mixed urban–rural setting as compared to our exclusively rural population.

Extrapolating a 0.76% incidence rate to the Rorya district, with a population of 354,490 inhabitants, suggests that 2694 people sustain an extremity fracture annually. Given the limited orthopedic capacity in Rorya, it is unsurprising that we find a dependence on TBSs to address this burden, reflected by the statistically significant majority of patients seeking TBS care. Despite their popularity, 32% attended a health facility, indicating a tendency to combine traditional and hospital care. Still, the low hospital attendance suggests that extremity fractures are largely underrepresented in hospital‐based data. The level of TBS patronage we found is higher than in studies conducted in other SSA countries. Studies reported 79.3% TBS attendance in Nigeria and 75% in Ghana, respectively [27, 28]. Solagberu found 23.1% of patients in Nigeria attended a TBS before presenting at the hospital [29], not accounting for those seeking TBS care after leaving the hospital [5].

Our survey discovered that travel time and costs to reach TBSs were comparable to those for dispensaries or health centers; over a third of respondents reported that even reaching hospital care was unaffordable. The similar level of access at the primary healthcare level offers an argument for increased communication between dispensaries/health centers and TBSs for patient triage and stabilization to select patients with more complex fractures for whom hospital referral is cost‐effective.

Furthermore, extremity fractures have substantial physical and financial consequences, with 62% of respondents reporting consequent inability to work as before or requiring help with transport and 50% experiencing reduced income. This often impacts the entire family, as caregiving responsibilities and income losses place additional burdens on other household members [30] and by extension the local community. In comparison, Nour et al. reported a higher rate of post‐injury disability (91.7%) and a similar challenge in affording basic necessities, such as food and rent (54.8%), in Cameroon [26].

Our finding that most fracture patients rely on TBSs highlights the unmonitored risk of adverse events from potentially inadequately managed complex fractures [31]. The major barriers to accessing timely orthopedic care for extremity fractures we noted include high hospital costs and poor health literacy. We concur with other authors that establishing communication and collaboration at the primary healthcare level could facilitate the triage of high‐risk patients and subsequent hospital referral, thereby reducing disability while respecting patient preferences for TBS when appropriate [13, 14, 32, 33]. Community health workers and village leaders could potentially play a crucial role in creating these local networks while also educating communities on safe fracture management [34]. To incentivize TBSs to be part of such a network, compensation for loss of income may be necessary, at least in the early stages. Finally, these findings support the urgent call for expanding affordable health insurance schemes to improve access to hospital care, ensuring that financial barriers do not prevent patients from receiving necessary medical attention, both in the acute and rehabilitative stage [35, 36]. Better coverage of orthopedic surgeons in rural areas could further help reduce costs related to health travel. A recent evaluation of Tanzania's National Surgical, Obstetric and Anesthesia Plan (NSOAP) highlighted the workforce shortage and gaps in service provision and capacity building as areas of attention [37, 38]. The abovementioned approaches for effective task‐sharing and referral system strengthening would align well with the national strategy in alleviating these problems. Similarly, including information on previous TBS attendance in recent digital initiatives in Tanzania, such as trauma registration and perioperative outcome recording [12, 39], could strengthen trauma data collection, a separate goal of Tanzania's NSOAP for quality monitoring.

## 5 Limitations

Recall bias could have led to overreporting or underreporting of fractures; we mitigated the risk of overreporting through the X‐ray verification. However, exclusion of deceased household members and reported older pediatric fractures with no radiographic evidence may yet have led to underreporting. Also, despite the pilot study to guide power calculation, our study was ultimately underpowered. A likely explanation is the X‐ray verification, which was employed in the main study, but not in the pilot study due to logistical issues. This underpowering limited the study's ability to perform extensive statistical analysis, for example into demographic factors influencing healthcare‐seeking behavior. Additionally, this survey did not evaluate whether fractures were open or closed, which would aid in interpretation of reported disability. Fracture type (i.e., open or closed) is not included in the SOSAS questionnaire, as a Gustilo–Anderson classification would be difficult to reliably obtain. Finally, future studies might expand the sample size to confirm our findings and explore a difference in physical and financial consequences of TBS attendance compared to hospital attendance.

## 6 Conclusions

We found an annual incidence rate of 0.76% for extremity fractures in Rorya district, Tanzania. Ninety‐five percent of these patients attend traditional bonesetters, whereas 32% seek hospital care. Primary reasons to seek out traditional treatment include increased affordability and perceived faster healing. However, around half of patients experience reduced capability to work with consequent loss of income. Establishing communication and cooperation between traditional bonesetters and the formal healthcare sector, augmented by improved access to effective fracture care, could improve hospital linkage for fracture patients and alleviate the burden of TBS‐related complications.

## Author Contributions

JB, TH, JO, KR, and AE conceived and designed the study. JB, AGW, JO, and TH decided on the sampling strategy. JB, JO, NW, and SO performed the survey and data collection. JB, AGW, and TH conducted the data analysis. JB, JO, SO, and AGW gave the survey team training. JB, JO, NW, and TH drafted the article. BC supervised the study and arranged facilities for survey team training. All authors critically reviewed the manuscript. All authors had access to the complete data. All authors contributed to, and endorsed, the final version submitted for publication.

## Ethics Approval and Consent to Participate

Ethical clearance was obtained on the 19th of June 2023, through the National Institute for Medical Research Tanzania, under reference number NIMR/HQ/R.8a/Vol.IX/4243. All participants provided written informed consent prior to enrollment in the study.

## Conflicts of Interest

The authors declare no conflicts of interest.

## Supporting information

Supplementary Material

## Data Availability

The data that supports the findings of this study are openly available in the Radboud Data Repository (RDR) at https://doi.org/10.34973/v4xj‐ct33, reference number [to be entered].

## References

[1] World Health Organization , Fact Sheet: Injuries and Violence, https://www.who.int/news‐room/fact‐sheets/detail/injuries‐and‐violence. Accessed, Jun 19, 2022.

[2] World Health Organization Fact , Sheet: Road Traffic Injuries, https://www.who.int/news‐room/fact‐sheets/detail/road‐traffic‐injuries. Accessed, Jun 19, 2022.

[3] A.‐M. Wu , C. Bisignano , S. L. James , et al., “Global, Regional, and National Burden of Bone Fractures in 204 Countries and Territories, 1990–2019: A Systematic Analysis From the Global Burden of Disease Study 2019,” Lancet Healthy Longevity 2, no. 9 (2021): e580–e592, 10.1016/S2666-7568(21)00172-0. 34723233 PMC8547262

[4] A. Premkumar , X. Ying , W. Mack Hardaker , et al., “Access to Orthopaedic Surgical Care in Northern Tanzania: A Modelling Study,” World Journal of Surgery 42, no. 10 (2018): 3081–3088, 10.1007/s00268-018-4630-x. 29696326

[5] S. K. Ruhinda , “Reasons for Patronage of Traditional Bone Setting as an Alternative to Orthodox Fracture Treatment A Case of Muleba District, Kagera Tanzania,” Huria: Journal of the Open University of Tanzania 27, no. 1 (2021): 29–44, 10.61538/huria.v27i1.867.

[6] M. J. H. Ariës , H. Joosten , H. H. J. Wegdam , and S. Van Der Geest , “Fracture Treatment by Bonesetters in Central Ghana: Patients Explain Their Choices and Experiences,” Tropical Medicine and International Health 12, no. 4 (2007): 564–574, 10.1111/j.1365-3156.2007.01822.x. 17445148

[7] B. R. Huber and R. Anderson , “Bonesetters and Curers in a Mexican Community: Conceptual Models, Status, and Gender,” Medical Anthropology 17, no. 1 (1996): 23–38, 10.1080/01459740.1996.9966126. 8757711

[8] S. Isaacs‐Pullins , M. Vaz , H. Murthy , D. Hughes , and K. J. Kallail , “A Qualitative Study of Traditional Bone Setters in South India: A Case Series,” Kansas Journal of Medicine 15, no. 3 (2022): 394–402, 10.17161/kjm.vol15.18580. 36467447 PMC9710505

[9] I. E. Abang , J. Asuquo , N. E. Ngim , et al., “Reasons for Patronage of Traditional Bone Setters,” Nigerian Journal of Surgery 22, no. 2 (2016): 102–106, 10.4103/1117-6806.188993. 27843274 PMC5013735

[10] T. Yempabe , A. Edusei , P. Donkor , A. Buunaaim , and C. Mock , “Traditional Bonesetters in Northern Ghana: Opportunities for Engagement With the Formal Health Sector,” Pan African Medical Journal 37 (2020): 248, 10.11604/pamj.2020.37.248.22420. 33552366 PMC7847210

[11] E. B. Card , J. E. Obayemi , O. Shirima , et al., “Practices and Perspectives of Traditional Bone Setters in Northern Tanzania,” Annals of Global Health 86 (2020): 1–8, 10.5334/AOGH.2878/METRICS/. 32587811 PMC7304448

[12] C. Osebo , T. Razek , J. Grushka , et al., “Impacting Trauma Care in Resource‐Limited Settings: Lessons Learned From Tanzania’s Web‐Based Trauma Registry Initiatives,” World Journal of Surgery 48, no. 10 (2024): 2515–2525, 10.1002/wjs.12333. 39267203

[13] A. Agarwal and R. Agarwal , “The Practice and Tradition of Bonesetting,” Education and Health 23, no. 1 (2010): 225, 10.4103/1357-6283.101508. 20589600

[14] A. B. Omololu , S. O. Ogunlade , and V. K. Gopaldasani , “The Practice of Traditional Bonesetting: Training Algorithm,” Clinical Orthopaedics and Related Research 466, no. 10 (2008): 2392–2398, 10.1007/S11999-008-0371-8. 18612711 PMC2584317

[15] C. Ezeanya‐Esiobu , The Case of Traditional Bonesetting and Orthopaedic Medical Curriculum (Springer, 2019), 81–95.

[16] A. U. Ekere and R. C. Echem , “Complications of Fracture and Dislocation Treatment by Traditional Bone Setters : A Private Practice Experience,” Nigerian Health Journal 11 (2011): 131–138.

[17] H. R. Sawe , J. A. Mfinanga , K. R. Mbaya , et al., “Trauma Burden in Tanzania: A One‐Day Survey of All District and Regional Public Hospitals,” BMC Emergency Medicine 17 (2017): 1–6, 10.1186/S12873-017-0141-6/TABLES/5. 29029604 PMC5640911

[18] National Bureau of Statistics Tanzania (2022) 2022 Population and Housing Census ‐ Administrative Units Population Distribution and Age and Sex Distribution Reports

[19] Chacha C. C. (2013) Rorya District Council Strategic Plan 2015‐2020

[20] A. Wiehl , I. Ray , D. Kammen , A. Hubbard , and D. Levine , “Nudging Towards Micro‐savings: A Step‐Wedge Experiment on LPG Adoption in Rural Tanzania,” AEA Randomized Controlled Trials, (2022) 10.1257/RCT.8465.

[21] Allaire JJ RStudio : Integrated Development Environment for R

[22] “SurveyCTO data collection platform,” SurveyCTO, accessed July 23, 2024, https://www.surveycto.com/.

[23] S. Gupta , U. Mahmood , S. Gurung , et al., “Burns in Nepal: A Population Based National Assessment,” Burns 41, no. 5 (2015): 1126–1132, 10.1016/j.burns.2014.11.012. 25523087

[24] R. T. Petroze , R. S. Groen , F. Niyonkuru , et al., “Estimating Operative Disease Prevalence in a Low‐Income Country: Results of a Nationwide Population Survey in Rwanda,” Surgery 153, no. 4 (2013): 457–464, 10.1016/j.surg.2012.10.001. 23253378

[25] Good Calculators Sample Size Calculator, https://goodcalculators.com/sample‐size‐calculator/. Accessed 23 Jul 2024

[26] F. M. Ahmed Nour , M. S. Tiee , R. A. Oke , et al., “Limb Injuries and Disability in the Southwest Region of Cameroon,” JAAOS Global Research & Reviews 7, no. 2 (2023): e2200148, 10.5435/jaaosglobal-d-22-00148. PMC9937092 36795867

[27] J. D. Ogunlusi , I. C. Okem , and L. M. Oginni , “Why Patients Patronize Traditional Bone Setters,” Internet Journal of Orthopedic Surgery 4 (2006).

[28] Abass A. F. “Patients Preference for Traditional Bonesetters in Northern Ghana.” (2015).

[29] Solagberu B. A. “Long Bone Fractures Treated by Traditional Bonesetters: A Study of Patients’ Behaviour.” Tropical Doctor 35, no. 2 (2005):106, 10.1258/0049475054036797 15970039

[30] C. N. Mock , S. Gloyd , S. Adjei , F. Acheampong , and O. Gish , “Economic Consequences of Injury and Resulting Family Coping Strategies in Ghana,” Accident Analysis & Prevention 35, no. 1 (2003): 81–90, 10.1016/S0001-4575(01)00092-6. 12479899

[31] N. O. Onyemaechi , W. N. A. Menson , X. Goodman , et al., “Complications of Traditional Bonesetting in Contemporary Fracture Care in Low‐ and Middle‐Income Countries: A Systematic Review,” Tropical Medicine and International Health 26, no. 11 (2021): 1367–1377, 10.1111/tmi.13662. 34309148

[32] Askew M. , Hinchman C. , Rayel I. , et al. “The Continued Role for Traditional Bonesetters in East and West Africa: An Expert Consensus Statement.” (2023).

[33] B. U Nwachukwu , I. C. Okwesili , M. B. Harris , and J. N. Katz , “Traditional Bonesetters and Contemporary Orthopaedic Fracture Care in a Developing Nation: Historical Aspects, Contemporary Status and Future Directions,” Open Orthopaedics Journal 5, no. 1 (2011): 20–26, 10.2174/1874325001105010020. 21270953 PMC3027080

[34] J. Binnerts , T. C. C. Hendriks , N. Buzugbe , et al., “Broad Support Among Stakeholders for Collaboration Between Traditional Bonesetters and Formal Healthcare: A Qualitative Study in a Resource‐Limited Setting,” Inquiry 62 (2025), 10.1177/00469580251325031. PMC11948548 40145286

[35] T. Yempabe , A. Edusei , P. Donkor , A. Buunaaim , and C. Mock , “Factors Affecting Utilization of Traditional Bonesetters in the Northern Region of Ghana,” African Journal of Emergency Medicine 11, no. 1 (2021): 105–110, 10.1016/j.afjem.2020.09.002. 33680729 PMC7910168

[36] A. M. Udosen , O. O. Otei , and O. Onuba , “Role of Traditional Bone Setters in Africa: Experience in Calabar, Nigeria,” Annals of African Medicine 5 (2006): 170–173.

[37] The United Republic of Tanzania Ministry of Health Community Development Gender Elderly and Children . National Surgical, Obstetric and Anesthesia Plan (NSOAP): 2018–2025 (2018), https://docs.wixstatic.com/ugd/d9a674_4daa353b73064f70ab6a53a96bb84ace.pdf.

[38] A. M. Hellar , L. Akoko , D. T. Jumbam , et al., “An Assessment of Progress and Challenges in the Implementation of the National Surgical, Obstetric, and Anesthesia Plan (NSOAP) in Tanzania,” East and Central African Journal of Surgery 29, no. 2 (2024): 15–22, 10.4314/ecajs.v29i2.4.

[39] C. Osebo , T. Razek , J. Grushka , et al., “Digitizing Operating Theater Data in Resource‐Limited Settings: Understanding Surgical Care Delivery Post‐implementation at Tanzanian Referral Hospital,” World Journal of Surgery 48, no. 8 (2024): 1873–1882, 10.1002/wjs.12239. 38850082

