# Supplementary material for: Incidence, Impact, and Healthcare‐Seeking Behavior for Extremity Fractures in Resource‐Limited Settings: A Household Survey in Rural Tanzania
Source: World J Surg. 2025 Apr 11;49(5):1368–76. doi: 10.1002/wjs.12540 (PMC12058443; doi:10.1002/wjs.12540)
Supplement: Supplementary file 1 — Supplementary Material [file WJS-49-1368-s001.docx]

Enumerator 5-day training program

## **Monday 26th**

Morning report room of Shirati KMT Hospital

10.00 - 10.30 Introductions

10.30 - 11.00 Explanation of the study aims and methodology

11.00 – 12.30 1^st^ session of supervised practice with the SOSAS and Random_HH_Fracture questionnaires, using mobile tablet and the SurveyCTO data collection software

12.30 - 13.30 Lunch
Adjustments to questionnaires, based on input from practice session

13.30 – 15.00 2^nd^ session of supervised practice

15.00 – 15.30 Explanation of 1^st^ individual practice
Room for questions

## **Tuesday 27th**

Home session

8.00 – 12.00 Individual practice with a relative at home
Data uploaded through SurveyCTO

12.00 – 17.00 Analysis by trainers of practice data for consistency and relevance
Adjustments to questionnaires, based on input from individual practice session

## **Wednesday 28th**

Morning report room of Shirati KMT Hospital

10.00 – 10.30 Update on results of the home session and any adjustments made

10.30 – 12.00 Round of training with supervised practice, focusing on adjusted segments

12.00 – 13.00 Lunch
 Adjustments to questionnaires, based on input from practice session

13.00 – 15.00 Round of training with supervised practice, focusing on adjusted segments

15.00 – 15.30 Explanation of 2^nd^ individual practice
Room for questions

## **Thursday 29th**

Home session

8.00 – 12.00 Individual practice with a random person from the enumerator’s village
Data uploaded through SurveyCTO

12.00 – 17.00 Analysis by trainers of practice data for consistency and relevance
Adjustments to questionnaires, based on input from individual practice session

## **Friday 30th**

Morning report room of Shirati KMT Hospital

10.00 – 10.30 Update on results of the 2^nd^ home session and any adjustments made

10.30 – 12.00 Round of training with supervised practice, focusing on adjusted segments of the survey

12.00 – 13.00 Lunch
 Adjustments to questionnaires, based on input from practice session

13.00 – 15.00 If necessary, round of training with supervised practice for those who need it, focusing on adjusted segments

15.00 – 15.30 Provision of schedule for pilot study, involving 50 households next week
 Signing of contracts
